# Cut-scores revisited: feasibility of a new method for group standard setting

**DOI:** 10.1186/s12909-018-1238-7

**Published:** 2018-06-07

**Authors:** Boaz Shulruf, Lee Coombes, Arvin Damodaran, Adrian Freeman, Philip Jones, Steve Lieberman, Phillippa Poole, Joel Rhee, Tim Wilkinson, Peter Harris

**Affiliations:** 1 University of New South Wales Australia, Sydney, Australia; 2 Cardiff University, Cardiff, UK; 3 University of Exeter, Exeter, UK; 4 University of Auckland, Auckland, New Zealand; 5 University of Texas, Austin, USA; 6 University of Otago, Dunedin, New Zealand

**Keywords:** Standard setting, MCQ, Angoff, Assessment

## Abstract

**Background:**

Standard setting is one of the most contentious topics in educational measurement. Commonly-used methods all have well reported limitations. To date, there is not conclusive evidence suggesting which standard setting method yields the highest validity.

**Methods:**

The method described and piloted in this study asked expert judges to estimate the scores on a real MCQ examination that they consider indicated a clear pass, clear fail, and pass mark for the examination as a whole. The mean and SD of the judges responses to these estimates, Z scores and confidence intervals were used to derive the cut-score and the confidence in it.

**Results:**

In this example the new method’s cut-score was higher than the judges’ estimate. The method also yielded estimates of statistical error which determine the range of the acceptable cut-score and the estimated level of confidence one may have in the accuracy of that cut-score.

**Conclusions:**

This new standard-setting method offers some advances, and possibly advantages, in that the decisions being asked of judges are based on firmer constructs, and it takes into account variation among judges.

## Background

Standard setting is a contentious topic in educational measurement. Commonly used methods all have reported limitations. To date there is no conclusive evidence suggesting which standard setting method yields the highest validity. A comprehensive review of standard setting in the book by G Cizek and M Bunch [1] provides an in-depth insight into a range of mechanisms by which judges’ perceptions of the desirable cut-scores are extracted and summarised. The axiom used by Cizek & Bunch is that ‘r*egardless of the procedure chosen, the standard setter will always need to involve people and judgments*’ [1]. This axiom is correct, but the range of both people and techniques involved in those judgements is wide and the variance across judges determining cut-scores is large [2–5]. Well-known is the Angoff method in which groups of judges estimate the proportions of hypothetical minimally competent examinees who would correctly answer each item. The mean of the proportions across all judges establishes the cut-score (henceforth: CS) [6]. Most other standard setting methods employ panels of experts who are asked to agree upon examination cut scores either by estimating the difficulty of the items, of the entire examination and/or estimating the acceptable pass/fail rates [1]. More advanced methods of this type provide the panellists with some psychometric parameters with which to facilitate or improve their judgement [7–10]. Alternative methods do not use panellists, but use the student examination marks to generate cut-scores, without any additional judgement [11–14]. The extent to which standards rely on, or are independent of, assessment data can vary even within implementations of the same method. To date no conclusive evidence is available to suggest which method is more accurate at identifying the cut-scores that best distinguish the competent from the incompetent examinees [4]). This is despite compelling evidence suggesting that whenever two or more different methods are applied to the same examination data the cut-score are almost always different [15–21].

A common reason for the lack of validity evidence for standard setting methods is that almost all the research uses observed data (examination marks) and judges’ perspectives to estimate cut-scores but information on the true abilities of students is rarely available. It is evident and well documented that different methods yield different cut-scores for the very same examination results with no evidence provided to suggest which method is superior to others [21]. If such data (the examinees’ ‘true’ ability) were available, there would be no need to set standards and cut-scores. Consequently, the quality of standard setting methods is commonly measured by the level of subjective agreement among judges, the reliability of the results, or the error of measurement of the yielded cut-scores [22–29].

A different approach for estimating the quality of standard setting methods and the accuracy (deviation from the ‘true’ cut-score, however it is defined) of the yielded cut-scores is to apply standard setting methods to simulated datasets where the ‘true ability’ is predetermined [30, 31]. This type of research does not measure natural or observed phenomena but rather measures only the accuracy of a standard setting technique under a defined set of assumptions; as part of an evidence-based approach [32–34]. Overall, it is a challenge to find a standard setting model applicable to observed data, yet providing a measure of accuracy beyond just the agreement of judges.

The current paper introduces a method which provides a partial solution to the abovementioned challenge. The main principles of this new method are: (1) it involves a panel of judges; (2) it assumes that the examination score that denotes a ‘clear pass’ or a ‘clear fail’ is more concrete and easier to estimate [35] than the concept of ‘the proportion of minimally competent examinees who would give a correct answer to each of the items’ [6]; (3) by measuring two cut-scores (minimum score for clear pass (cP) and maximum score for clear fail (cF)) it doubles the number of data points, hence may provide more reliable cut-scores [36]; (4) the examination cut-score means that if the examination were free of measurement- errors, every performance level below that cut-score indicates incompetence and any performance level at or above the cut-score indicates competence. Consequently, one could be classified as either competent or incompetent but never both or neither and ultimately a borderline mark/score is only given when the measurement is not accurate enough to provide a decisive pass/fail mark; and (5) the examination cut-score is based on 95% confidence intervals (95%CI) calculated from the distributions of judges’ scores for cF and cP. The distributions are normalised, and the cut point is at the optimal interface between the two distributions with the same z-scores for cF and cP. In other words, this new method yields a cut-score which is derived from the clear criteria estimates of the scores representing ‘clear pass’ and ‘clear fail’, which have been demonstrated to be reliable reference criteria [37].

### Application of the new method

In a usual Angoff procedure, each person in a panel of judges reviews the examination, and estimates the proportion of minimally competent examinees likely to give the correct answer to each of the items. The scores across items and judges are then averaged to determine the cut-score. In our new method, the judges may also review each item; however, this is done to allow them to gauge the overall impression of the exam difficulty. With their knowledge of the examination difficulty, the student level and the curriculum, the judges answer the following two questions for the examination as a whole:

1. What would be the lowest score that indicates the examinee is without any doubt, clearly competent in the topics assessed?
2. What would be the highest score that indicates the examinee is without any doubt, clearly incompetent in the topics assessed?

The only data used in this method are the scores independently given by the judges in response to the above two questions.

#### Calculating the cut-score

Each judge provides two scores:

- L = the highest score indicating the examinee is clearly incompetent.
- H = the lowest score indicating the examinee is clearly competent.

From the collated scores (L & H), means of L and H (X_L_ and X_H_ respectively) and standard errors of the means (SE_L_ and SE_H_ respectively) are calculated.

The following equation is used to identify the Z score (Z) which would apply to both confidence intervals of X_L_ and X_H_ when they interface.

1 $$ {\mathsf{Z}}^{\ast }\ {\mathsf{SE}}_{\mathsf{L}}\kern0.5em +{\mathsf{Z}}^{\ast }\ {\mathsf{SE}}_{\mathsf{H}}=\mathsf{H}-\mathsf{L} $$

From (1) that we extract the Z:

2 $$ \mathsf{Z}=\left(\mathsf{H}-\mathsf{L}\right)/\left(\ {\mathsf{SE}}_{\mathsf{L}}\kern0.5em +{\mathsf{SE}}_{\mathsf{H}}\right) $$

Then the cut-score is where L + Z* SE_L_ = H- Z* SE_H_.

A Z-score table is then used to identify the statistical confidence of the cut-score. In other words, this indicates the level of confidence one may have in that cut-score being correct.

**Fig. 1:**
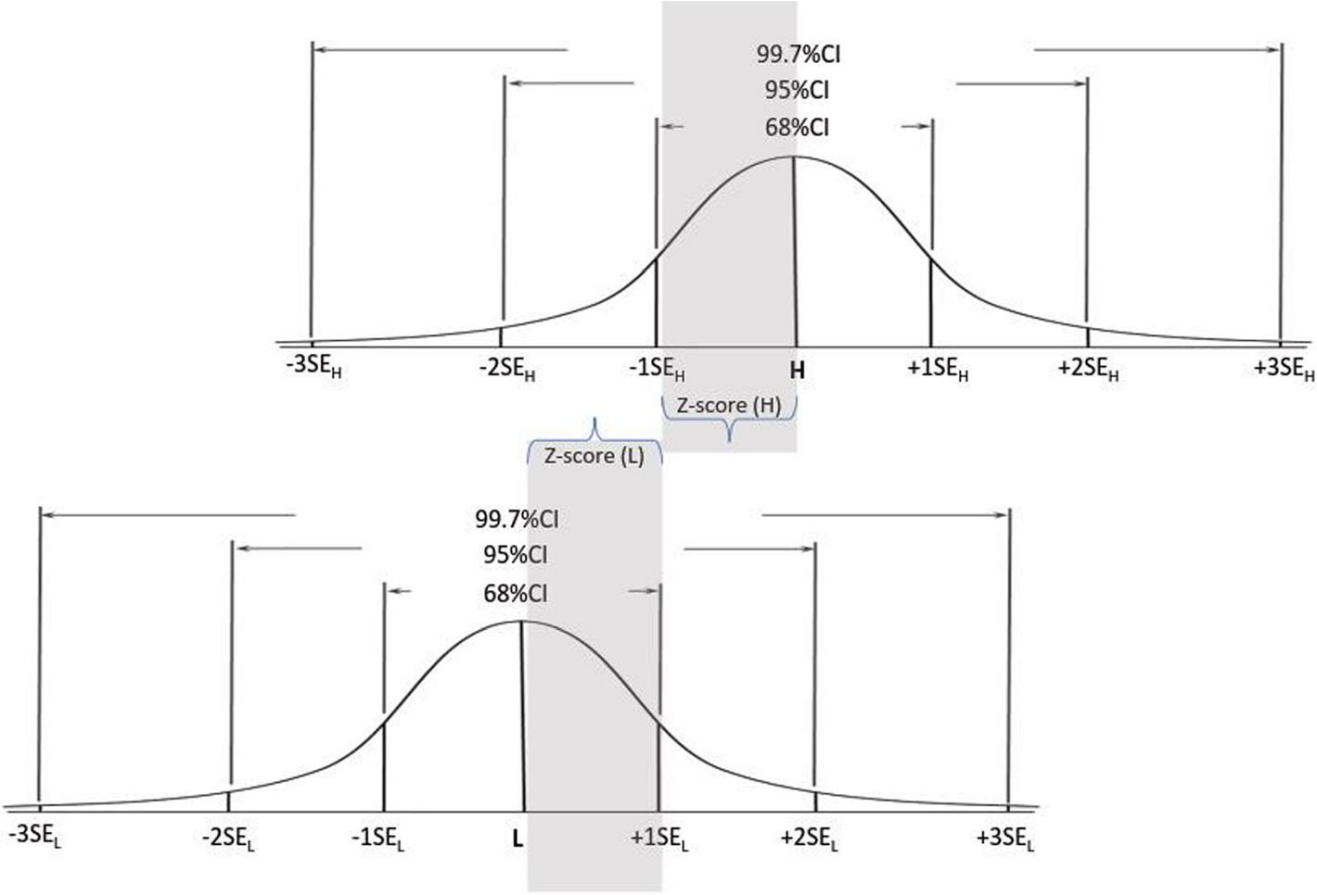
Setting the cut score at the confidence level of 68%

**Fig. 2:**
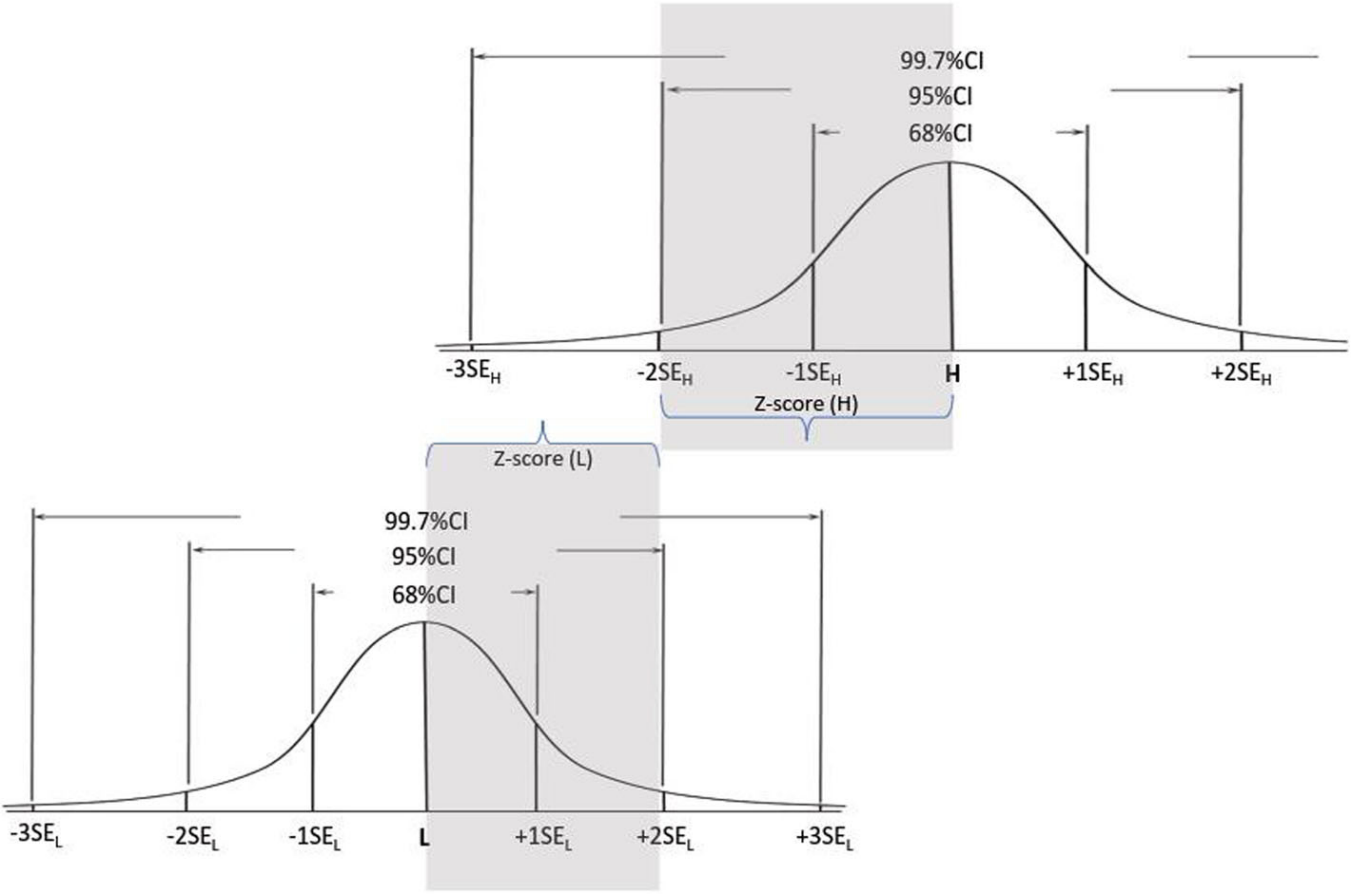
Setting the cut score at the confidence level of 95%

**Fig. 3:**
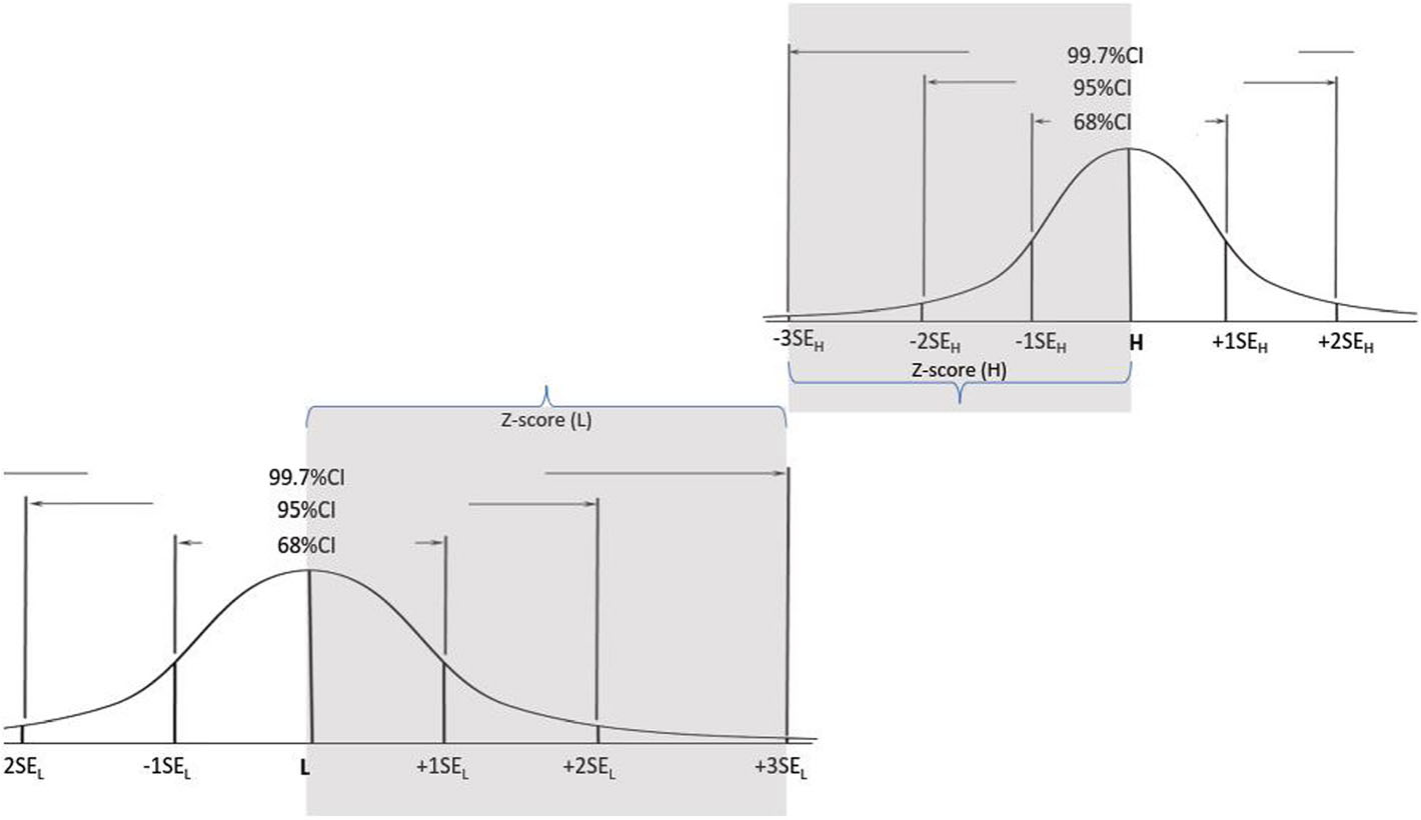
Setting the cut score at the confidence level of 99.7%

A demonstration of the method is presented below in Figs. 1, 2 and 3.

Figures 1 demonstrates a hypothetical situation when: SE_L_ = SE_H_; and 1SE_L_+ 1SE_H_ = H – L. In this demonstration, SE sizes determine that there is 68% confidence that a score below the cut-score (CS) indicates incompetence and a score above the CS indicates competence. The CS is placed in the optimal location where the confidence that a score below it indicates incompetence, and a score above it indicate competence are equal (=68%). In this example both L and H are placed within 1 SE (SE_L_ and SH_H_ respectively) from the CS; and since SE_L_ = SE_H_, CS -L = H-CS that is the CS is placed within the same distance from L and H.

Figures 2 demonstrates a hypothetical situation when the SEs are not equal i.e. SE_L_ < SE_H_; and the distance of the L and H from the CS is 2 SEs (2SE_L_+ 2SE_H_ = H – L). In this example the SE sizes determine the CS is placed in the optimal location where the confidence that a score below it indicates incompetence, and a score above it indicate competence are equal (=95%). In this example, both L and H are placed within 2 SE (SE_L_ and SH_H_ respectively) from the CS. However, unlike in Fig. 1, in this example SE_L_ < SE_H,_ thus CS -L < H-CS, which then places the CS closer to L than to H. In this example it is clear that the examiners reached greater agreement about the value of L than about the value of H. It is noteworthy that although the CS is not in the middle between L and H, it is still in the optimal location which yields the same confidence for competence and incompetence for scores above and below (respectively) the CS.

The third example (Fig. 3) demonstrates another hypothetical situation when the SEs are not equal, i.e. SE_L_ > SE_H_, and the distance of L and H from the CS is 3 SEs (3SE_L_+ 3SE_H_ = H – L). In this example the SE sizes determine that the CS is placed in the optimal location where the confidence that a score below it indicates incompetence, and a score above it indicates competence are equal (=99.7%). Similar to previous examples (Figs. 1 & 2), in this example, both L and H are each placed within the same number of SEs (3SE_L_ and 3SH_H_ respectively) from the CS. However, in this example (Fig. 3) SE_L_ > SE_H,_ thus CS -L > H-CS, which then places the CS closer to H than to L. In this example it is clear that the examiners reached greater agreement about the value of H than about the value of L. Also, although the CS is not in the middle between L and H, it is still in the optimal location which yields the same confidence for competence and incompetence for scores above and below (respectively) the CS.

Of note is that the CS is always located in a place that equates the confidence that a score just under the CS indicates incompetence with the confidence that a score just above the CS indicated competence. The new method does not assume that the agreement among the assessors would be the same about thresholds for clear pass and clear fail; and no previous study supporting such an assumption (of equity) was identified.

This new method assumes that the judges’ L’s and H’s are normally distributed. This assumption is reasonable should the judges composition be balanced [30]. If judges’ composition is not balanced then their judgement would be skewed irrespective of the method used, whether it was with this new method, Angoff, Abel, Bookmark or any other. Possible, yet partial remedies for heavily skewed scores are either to remove the extreme scores or applying bootstrapping to calculate robust SEs [38, 39]. These techniques may be useful also for other standard setting methods relying on SEs [26, 29, 39, 40]. Certainly, increasing the number of judges is also likely to normalise the judges’ L and H scores’ distribution.

This paper demonstrates and discusses the application and the feasibility of this new method.

## Methods

For this feasibility study, we used 20 multiple choice questionnaire (MCQ) items taken from the final written examination used for medical students at an Australian university. This examination is set at the medical programme graduate level and the items were placed into a web-based survey.

Experienced clinical teachers who were familiar with the expected level of medical programme graduates were invited to participate in the study. The respondents provided information on their level of training, gender and age, as well as responses to the following questions:

1. What would be the lowest score for the entire examination that would indicate that the examinee is without any doubts, clearly competent in the topic assessed?
2. What would be the highest score for the entire examination that would indicate that the examinee is without any doubts, clearly incompetent in the topic assessed?

Finally, respondents were asked to suggest a cut-score for the entire examination.

The study was approved by Human Research Ethics Advisory (HREA) Panel G: Health, Medical, Community and Social ref. # HC16181. Participants consented to complete the survey and then review the draft paper containing collated anonymised data.

## Results

Seventeen participants participated in the questionnaire. This is an acceptable size for traditional Angoff processes applied in medical education [41–44].

Table 1 shows the means and standard errors of the means of judges’ scores for L and H, as well as their estimates for the exam as a whole.

**Table 1:** Descriptive statistics of judges’ scores

	Min Competent (H)	Max Incompetent (L)	Suggested Cut-score
*N*	17	17	17
Mean^a^	65.35	62.65	60.47
Std. Error of Mean	3.193	5.609	2.756
Std. Deviation	13.16	23.12	11.36

^a^Scores are %

The cut-score was calculated as per the description above with the H and L data from Table 1 in inserted into the equations.

Using eq. (1) Z* SE_L_ + Z* SE_H_ = H-L; Z*5.609 + Z*3.193 = 65.35–62.65 we calculated **Z = 0.307** .

Consequently, using the data derived from L, the cut-score decided by the panel is L + Z* SE_L_ = 62.65 + 0.307*5.609 = **64.37**.

The cut-score using the data derived from H is obviously identical H- Z* SE_H_ = 65.35–0.307*3.193 = **64.37**.

With Z = 0.307, the *p* value = 0.38 (based on a Z-table). This means there is only a 25% level confidence (areas under the normal curve around L, from the CS upwards =12.5%; and under the normal curve around H from the CS downwards =12.5% [45]) that the cut-score of 64.37 correctly distinguishes between pass and fail based on judges’ reports. A cut-score > 64.37 would *increase* the confidence that a score above it is not fail but would *decrease* the confidence that a score below it is not pass and vice versa. In the absence of external valid information about student abilities, there is no other way to increase the confidence of both.

There is another potential way to calculate the 95%CI for the cut-score, which is relevant only when the |Z-score| < 1.96, as in these results (Z = 0.307). In this case the 95%CI of the L and the H are used. Given that one could be either competent or incompetent but cannot be neither or both it is clear that (H- 1.96 SE_H_) is the lowest 95%CI boundary of the score that would be acceptable by the examiners as clear pass. Thus any score < (H- 1.96*SE_H_) must indicate failure. Similarly, L+ 1.96*SE_L_ is the highest 95%CI boundary of the score that would be acceptable by the examiners as clear pass. Thus any score < L+ 1.96 SE_L_ must indicate failure. Consequently, the 95%CI of the ANGOFF 2.0 given |Z-score| < 2 is: L+ 1.96 SE_L_ to H- 1.96 SE_H_. Using the results presented in Table 1 the 95%CI of the cut-score between 59.09 and 73.64. It is important to note that the cut-score may not necessarily equal the mid-point between two boundaries of the 95%CI: the cut-score is 64.37 whereas the mid-point of the ANGOFF 2.0 95%CI is (59.09 + 73.64)/2 is 66.37. The reason is that the *95%CI of the cut-score* is derived from *two different and independent variances* (the L and H scores were obtained independently. Nonetheless from the data in this study, the cut-score of 64.37 was only slightly different from the mid-point between L and H: (65.35 + 62.65)/2 = 64.00, but very different from the mean of the cut-scores as suggested by the very same judges (60.47).

## Discussion

This study describes a new and feasible way to determine cut-scores using a panel of judges. It is different from Angoff and modified Angoff methods in one major way. The Angoff method and its variants ask judges about the proportion of minimally competent examinees who would give a correct answer to *each of the items*. This is a complex cognitive process that requires the judges to make several decisions: identify what the minimally competent examinee is; and the proportion of such hypothetical examinees that would correctly answer each item. These decisions are made on relatively vague criteria which may leave standard setters unsure of the standard’s reliability. The means of the proportions of this hypothetical minimally competent examinees who would correctly answer each item then determine the cut-score [6, 46]. Furthermore, the empirical association between *proportions of examinees* and an *examination cut-score* has not been discussed in the literature, thus can at best be an arbitrary mechanism [1, 47].

By contrast, our proposed method uses the judges’ estimates of level of performance. The judges may estimate the proportion of examinees who would correctly answer each item, but this exercise is used only to facilitate judges’ judgements and impressions of the examination as a whole. These proportions derived *are not used* to calculate the cut-score. The new method directly asks judges to determine cut-scores for the whole examination, using practical concepts of ‘clear pass’ and ‘clear fail’ without referring to any hypothetical concept. As discussed above, having concrete points of reference or principles may enhance the accuracy of the determined cut-scores [48, 49].

We found the cut-score determined by this new method was different from a cut-score yielded from a direct question asking about the desirable cut-score for an examination (Table 1). So which cut-score is more trustworthy? It has been demonstrated that the use of the categories of ‘clear pass’ and ‘clear fail’ better distinguish between competent and incompetent examinees than the categories of ‘pass’ and ‘fail’ [37]. It is not unreasonable to conclude that a cut-score that uses the ‘clear pass’ and ‘clear fail’ as reference is more accurate than a cut-score that uses estimates of competence within the borderline range (e.g. estimations based on *minimally competent examinees*). Recent debate has suggested three mechanisms that explain variances among assessors’ judgements: (1) they apply assessment criteria incorrectly; (2) there are fundamental limitations in human cognition; and (3) assessors may be meaningfully idiosyncratic and can make sense of complex and highly contextual situations which then lead to different outcomes, all of which are acceptable [50]. C St-Onge, M Chamberland, A Lévesque and L Varpio [51] echoed these assertions and suggested that it is mostly about a balance between external and internal/personal sources of information that impact assessors. The ImpExp model [52], for example, provides a detailed explanation of that process of responses to questions which overall indicates that variance among Angoff judges is unavoidable.

Our new method addresses the limitation of judges’ variance in a number of ways. First the method asks the judges to make judgements about what is clear (clear pass and clear fail) rather than what is vague (probability of correctly answering an item by a minimally competent examinee). What is a clear pass and what is a clear fail can be more easily agreed among assessors as these are based on principles that do not frequently change [49]. The data from the current study provide evidence for a difference between ‘principle’ based decisions (clear pass / clear fail) and decisions based on a vague reference (an ‘*estimate the proportion of hypothetical minimally competent examinees who would correctly answer each item*’ [6]). Based on data generated from the same judges for the same set of items, the new method cut-score was 64.37 but the suggested cut-score when asked directly was 60.47, which is even lower than the L (62.65). It is also noted when using judges in standard setting processes variability across judges’ cut scores is not a ‘random error’ in the sense of a typical measurement model [27]. That variability may be related to a range of biases derived from judges characteristics, opinions, expertise as well as other factors which should be *considered* rather than *minimised* [27, 30, 53, 54].

So what is the preferred cut-score? We believe that the new method cut-score is more trustworthy, firstly, as it is derived from mathematical principles, whereas the directly suggested cut-score is based on an overall impression of the examination difficulty and provides a less defensible cut score. Since asking examiners why they made each decision was not within the scope of the study this is a topic for future studies.

Secondly, the new method uses two points of reference (clear pass & clear fail) compared to only one (the expected level of the minimally competent examinee) used by the traditional and modified Angoff methods. Overall the traditional Angoff and its variants use more data but can be expensive in time and money, whereas the new method uses less data but is quick and inexpensive.

Third, the new method considers two independent variances (Var (L) and Var(H)). Table 1 demonstrates that these variances were different (Var(L) > Var(H)), which indicates that the judges agreed more closely about what constitutes clear pass than what constitutes clear fail. A recent study demonstrated that even one extreme examiner may impact the pass/fail decisions [55]. The new method provides an inherent moderating mechanism for extreme judges as the cut-score is determined not only by the L and the H but also by the related variances. The use of two different variances determines a cut-score which is not necessarily at the mid-point between the H and the L (64.37 vs. 64.00 respectively). We suggest that this is a preferable outcome since the mid-point between the H and the L does not consider differences in agreement among judges.

Last but not least, the new method optimises the balance between the false positive and false negative and estimates the confidence that the cut-score is correct. The data used in the current study demonstrate that the confidence was relatively low (25%). Nonetheless, this is the most balanced cut-score reachable given this particular examination and judges (the same z-score for clear pass and clear fail). Similar to many other Angoff methods [30, 56] the level of confidence in the cut-score may increase as the number of judges increase. Nevertheless, the level of confidence in the cut-score should not be of concern since although the closer the H and the L are to each other the smaller the confidence is; a close gap between the L and the H is a desirable and defensible outcome as it is an indication that the judges believe the examination has a high discrimination value.

## Conclusions

This feasibility study demonstrates how a revised Angoff method could generate a defensible cut-score. The demonstration was made on a limited sample of 17 judges examining 20 items only. Nonetheless the mathematical basis of the method is robust and thus suggests that it may be a feasible and defensible method for setting examination cut-scores. We anticipate that it would be applicable to most types of examinations which have an examination score and require a defensible cut-score. These could be examinations containing MCQs, OSCEs, short answers questionnaires etc. Further research, however, is required to identify how the new method compares to other methods and whether there are any limitations which have yet not been identified.

## Abbreviations

cF: Clear fail
cP: Clear pass
H: High boundary of 95% confidence interval of the mean
L: Low boundary of 95% confidence interval of the mean
MCQ: Multiple choice questionnaire
SD: Standard deviation
SE: Standard error
Var: Variance
Z: Z-score
